# Advancing sustainable healthcare: a concept analysis of eco-conscious nursing practices

**DOI:** 10.1186/s12912-024-02197-0

**Published:** 2024-09-16

**Authors:** Marwa Mamdouh Shaban, Majed Awad Alanazi, Huda Hamdy Mohammed, Fatma Gomaa Mohamed Amer, Hla Hosny Elsayed, Mohammed ElSayed Zaky, Osama Mohammed Elsayed Ramadan, Mohamed Ezzelregal Abdelgawad, Mostafa Shaban

**Affiliations:** 1 Faculty of Nursing, Cairo University, Cairo, Egypt; 2 College of Nursing, Jouf University, Sakaka, Saudi Arabia; 3 PhD Candidate- Faculty of Nursing- Cairo University, Cairo, Egypt; 4 Faculty of Nursing, Ain Shams University, Cairo, Egypt; 5 Faculty of Nursing, Alexandria University, Alexandria, Egypt

**Keywords:** Eco-conscious nursing, Environmental sustainability, Sustainable Healthcare practices, Nursing education, Environmental stewardship

## Abstract

**Background:**

As the healthcare sector grapples with its environmental footprint, the concept of Eco-conscious Nursing emerges as a pivotal framework for integrating sustainability into nursing practice. This study aims to clarify and operationalize Eco-conscious Nursing, examining its attributes, antecedents, consequences, and providing operational definitions to guide future research and practice.

**Methods:**

Utilizing a systematic literature review across PubMed, Google Scholar, and CINAHL Ultimate, this study identifies and analyzes existing theories, frameworks, and practices related to eco-conscious nursing. Through conceptual analysis, key attributes, antecedents, and consequences of Eco-conscious Nursing are delineated, leading to the formulation of comprehensive operational definitions.

**Results:**

The study reveals Eco-conscious Nursing as a multifaceted concept characterized by environmental stewardship, sustainable healthcare practices, and a commitment to reducing the ecological impact of nursing care. Operational definitions highlight the role of education, awareness, and institutional support as antecedents, with improved environmental health and sustainable healthcare outcomes as key consequences.

**Conclusion:**

Eco-conscious Nursing represents a crucial ethos for the nursing profession, emphasizing the necessity of sustainable practices within healthcare. The operational definitions provided serve as a foundation for embedding eco-conscious principles into nursing, addressing the urgent need for sustainability in healthcare settings. Future research should focus on the empirical application of these definitions and explore the economic and cross-cultural dimensions of eco-conscious nursing.

## Introduction

In the realm of healthcare, the burgeoning emphasis on sustainability has sparked a transformative movement towards environmentally conscious practices [1–3]. This shift recognizes the substantial environmental footprint left by healthcare operations, from extensive waste generation to significant energy consumption and greenhouse gas emissions [4]. As stewards of health, the healthcare sector is reevaluating its impact on the planet, aiming to reconcile the delivery of exceptional care with the imperative to preserve environmental integrity for future generations [5]. This holistic approach to healthcare not only addresses immediate health needs but also acknowledges the long-term implications of environmental health on overall well-being [6]. The intersection of healthcare and environmental stewardship underscores the urgent need for practices that ensure the health of the patient does not come at the expense of the planet’s health [7, 8].

Nurses, who form the backbone of healthcare services worldwide, are pivotal to this paradigm shift [9]. Historically, nursing has been intricately linked with environmental health, drawing from foundational principles laid down by pioneers like Florence Nightingale, who underscored the importance of sanitation, fresh air, and clean water [10]. Today, the nursing profession is expanding its scope to include environmental sustainability as a core component of healthcare delivery [11]. Nurses are increasingly recognized as essential agents of change, capable of influencing sustainable practices within healthcare settings [12]. Their close proximity to patients and integral role in healthcare operations positions them uniquely to advocate for and implement eco-friendly practices, embedding sustainability into the very fabric of healthcare [13].

However, the integration of eco-conscious principles into nursing practice is not without its challenges. Barriers such as a lack of awareness, insufficient training in sustainable practices, and entrenched systemic obstacles often hinder progress [14]. Despite these challenges, the transition towards eco-conscious nursing practices presents a myriad of opportunities for enhancing both environmental and patient outcomes [14]. For instance, effective waste management and energy-efficient practices not only mitigate environmental harm but also have the potential to reduce hospital-acquired infections and lower healthcare costs, illustrating the dual benefits of eco-conscious nursing [15].

The concept of eco-conscious nursing has emerged as a vital response to these challenges and opportunities, signifying a commitment to the principles of sustainability within the nursing profession [16]. This nascent concept, which seeks to harmonize nursing care with environmental stewardship, is still in the process of being defined and operationalized [14]. The aim is to develop a clear and actionable framework that nurses can adopt, ensuring that their work contributes positively to the health of the planet. By refining and embracing the concept of eco-conscious nursing, the profession can make significant strides towards a more sustainable and ethically responsible practice.

In support of this movement, notable nursing theorists have emphasized the importance of environmental considerations. Fawcett (2022) and Fawcett (2024) have articulated the evolution of nursing’s metaparadigm to include environmental and cultural components, highlighting the growing recognition of environmental health within the profession. Additionally, blogs on nursology.net provide valuable insights into nursing’s engagement with climate change and sustainability issues [17, 18]​.

The positions of major health organizations further underscore the urgency of this transition. The World Health Organization (WHO), the International Labour Organization (ILO), and the International Council of Nurses (ICN) have all published reports emphasizing the critical nature of addressing climate change and its impacts on health [19]. Recent international conferences on climate change, such as COP 28 in 2023, have highlighted the global health emergency posed by environmental degradation and called for immediate and sustained action​ [20].

The journey toward fully integrating eco-conscious principles within nursing practices necessitates a collaborative effort that spans beyond individual nurses to encompass healthcare institutions, educational bodies, and policy frameworks [21]. This collaborative approach involves the creation and adoption of policies that support sustainable practices, the development of nursing curricula that include environmental health, and the establishment of healthcare infrastructure that prioritizes sustainability [22]. Educational initiatives are particularly crucial, as they equip future nurses with the knowledge and skills needed to implement eco-conscious practices effectively [23]. Moreover, research plays a vital role in this transition, offering evidence-based strategies to mitigate the environmental impact of healthcare operations [24]. Through a concerted effort across these domains, the nursing profession can lead by example, demonstrating how healthcare can contribute to environmental sustainability without compromising the quality of patient care [25].

The movement towards eco-conscious nursing is a critical step in the evolution of healthcare, reflecting a growing recognition of the interconnectedness of human and environmental health [26]. By integrating sustainable practices into nursing, the healthcare sector can significantly reduce its environmental footprint, setting a precedent for responsible and ethical care delivery [27]. This shift not only aligns with global health priorities, such as the United Nations Sustainable Development Goals but also empowers nurses to take a proactive role in shaping a healthier and more sustainable future. As this concept continues to evolve, it will undoubtedly pave the way for innovative practices that ensure the well-being of patients and the planet alike [28].

## Methodology

**Fig. 1:**
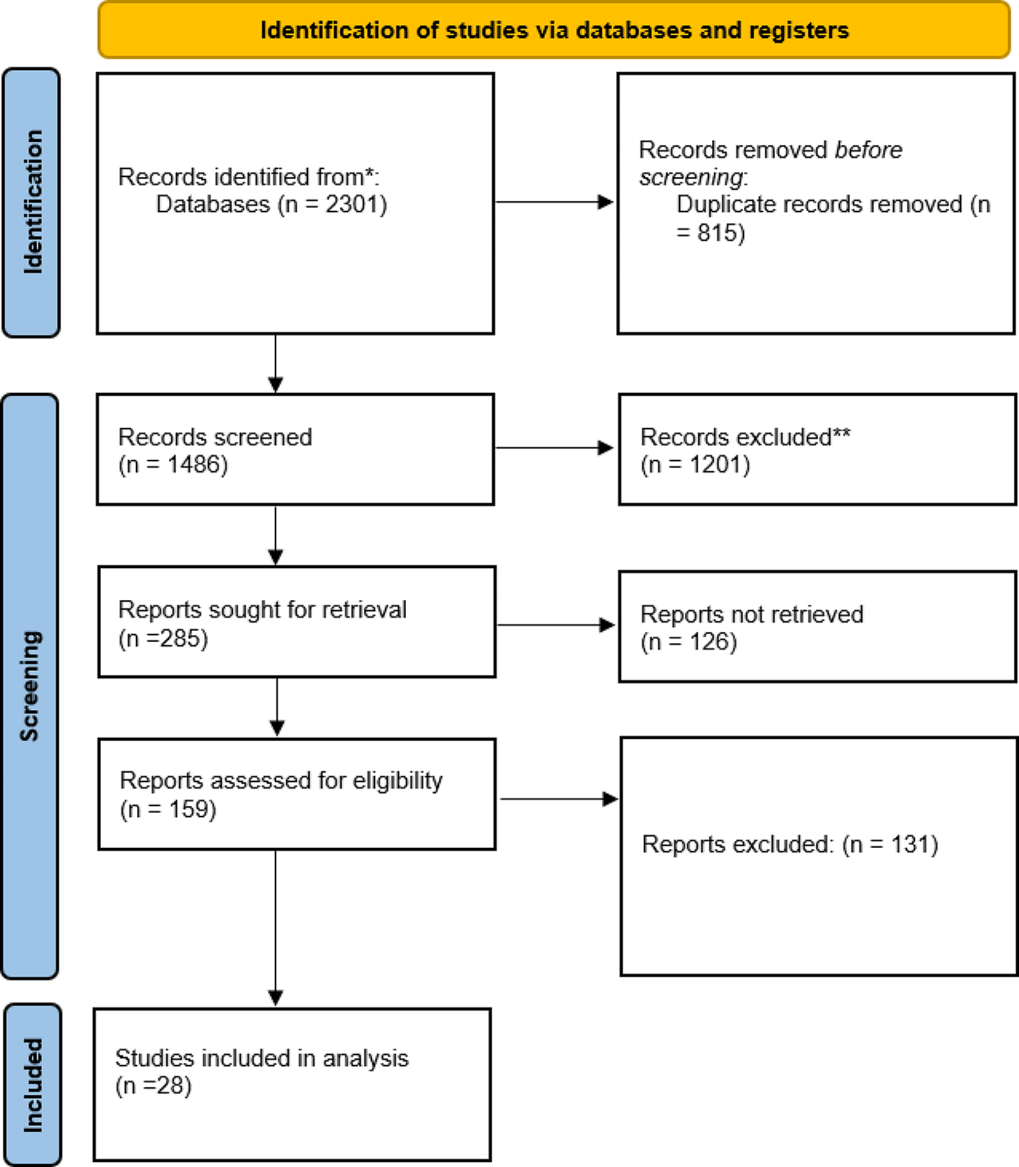
PRISMA Flow Diagram of the Literature Search Process

This concept analysis of eco-conscious nursing employed Rodgers’ evolutionary method, a well-established framework in nursing research that facilitates the clarification and operationalization of complex concepts [29]. The following steps were taken to ensure a comprehensive and systematic approach:

1. Theoretical Framework:

- The conceptual analysis was guided by Rodgers’ evolutionary method, which views concepts as dynamic and context-dependent, evolving over time based on the interplay of various factors. This method involves identifying the attributes, antecedents, and consequences of a concept through systematic literature review and analysis.

2. Literature Search Strategy:

- A comprehensive literature search was conducted across multiple databases, including PubMed, Google Scholar, and CINAHL Ultimate. The search incorporated a mix of specific Medical Subject Headings (MeSH) and keywords pertinent to the intersection of environmental sustainability and nursing practice. The chosen MeSH terms included ‘Environmental Health,’ ‘Sustainability,’ ‘Healthcare Waste,’ ‘Energy Conservation,’ ‘Nursing Care,’ ‘Nursing Practice,’ ‘Green Healthcare,’ and ‘Eco-friendly Practices.‘
- The search strategy was structured using Boolean operators to broaden the scope: ((((((((Environmental Health) OR (Sustainability)) OR (Healthcare Waste)) OR (Energy Conservation)) OR (Nursing Care)) OR (Nursing Practice)) OR (Green Healthcare)) OR (Eco-friendly Practices)) AND (Nursing).

3. Selection Criteria:

- A comprehensive search across multiple databases, including PubMed, Google Scholar, and CINAHL Ultimate, identified 2301 records. After removing 815 duplicates, 1486 records were screened, resulting in the exclusion of 1201 irrelevant records. From the remaining, 285 reports were sought for retrieval, but 126 could not be accessed. Of the 159 reports assessed for eligibility, 131 were excluded due to various reasons, such as not meeting the inclusion criteria or irrelevance. Ultimately, 28 studies were included in the qualitative synthesis and analysis, providing the necessary data to support the concept analysis of eco-conscious nursing, following the PRIMSA flow diagram, the selection was shown in Fig. 1.
- Inclusion criteria were: peer-reviewed articles, publications in English, and studies directly related to eco-conscious nursing practices. Exclusion criteria included non-peer-reviewed articles, publications in languages other than English, and studies not directly addressing eco-conscious nursing.

4. Analysis Process:

- The final selection of articles underwent an in-depth review to distill data pertinent to eco-conscious nursing practices, focusing on identifying the attributes, antecedents, and consequences of the concept. Abstracts and full texts were scrutinized where necessary to ensure a thorough understanding and synthesis of the prevailing knowledge landscape.
- Key themes were identified, including the integration of sustainability into nursing education and practice, the role of nurses in promoting environmental health, and the challenges and opportunities inherent in shifting towards more sustainable healthcare practices.

5. Quality Criteria:

- The quality of selected publications was assessed based on their methodological rigor, relevance to the concept of eco-conscious nursing, and contribution to understanding the attributes, antecedents, and consequences of the concept. Publications were included if they provided substantial empirical evidence, theoretical insights, or practical examples of eco-conscious nursing practices.

6. Temporal Scope:

- The review included publications from the past two decades to capture the evolution of eco-conscious nursing and its current state in the context of contemporary healthcare practices. This temporal scope ensured the inclusion of both foundational studies and recent advancements in the field.

### Uses of the concept

The concept of “Eco-conscious Nursing” plays a pivotal role in a variety of contexts within healthcare, emphasizing the integration of environmental sustainability principles into nursing practice. As healthcare systems globally grapple with their environmental impact, eco-conscious nursing emerges as a vital strategy aimed at reducing waste, conserving resources, and promoting a sustainable approach to patient care and facility operations [30]. This concept, broadly defined, encompasses practices that seek to minimize the environmental footprint of healthcare activities, advocating for environmentally responsible decision-making in clinical settings [31, 32]. This concept, broadly defined, encompasses practices that seek to minimize the environmental footprint of healthcare activities, advocating for environmentally responsible decision-making in clinical settings.

In clinical practice, the scope of eco-conscious nursing encompasses a diverse range of activities aimed at promoting environmental sustainability within healthcare settings. This involves the adoption of energy-saving measures such as the utilization of energy-efficient lighting and heating, ventilation, and air conditioning (HVAC) systems, which significantly reduce the carbon footprint of healthcare facilities [33]. Additionally, eco-conscious nursing advocates for the reduction of medical waste through strategies such as recycling programs, the proper segregation of waste, and the use of biodegradable products wherever possible [34]. The push towards the use of sustainable materials and resources further underscores the profession’s commitment to minimizing environmental harm. This includes advocating for the procurement of sustainably sourced medical supplies and the integration of eco-friendly products into daily nursing practice [24, 32]. These practices highlight the defining attribute of sustainability integration, reflecting the profession’s commitment to minimizing environmental harm.

These eco-conscious practices extend beyond the immediate benefits of environmental conservation, offering profound implications for patient care and staff wellbeing [35]. The implementation of green practices within clinical settings has been shown to enhance the healing environment, contributing to faster patient recovery times and improved mental health outcomes [36]. The presence of natural elements and increased access to outdoor spaces can significantly reduce stress levels among patients and staff alike, fostering a more positive and restorative hospital experience [37]. Moreover, by reducing exposure to harmful chemicals and pollutants through the adoption of non-toxic cleaning agents and sustainable materials, eco-conscious nursing also contributes to a safer, healthier workplace [38]. This holistic approach not only benefits the environment but also promotes a culture of health and wellbeing within healthcare institutions, demonstrating that sustainable practices in nursing can lead to improved health outcomes and enhanced staff satisfaction [39]. This advocacy aspect underscores the defining attribute of environmental advocacy, highlighting nurses’ roles as proactive agents of change within and beyond healthcare settings.

Beyond the immediate healthcare environment, eco-conscious nursing plays a crucial role in public health and community engagement. Nurses, leveraging their trusted position in society, are uniquely positioned to advocate for environmental health, educating patients and communities about the health impacts of pollution, climate change, and other environmental hazards [40]. This role includes participating in policy advocacy, pushing for regulations and policies that support environmental sustainability in healthcare and the wider community [41].

Educational initiatives represent a pivotal aspect of eco-conscious nursing, offering a foundational approach for embedding sustainability within the core of nursing education [42]. By integrating principles of environmental stewardship into nursing curricula, educational institutions are preparing future nurses to not only excel in clinical competencies but also to actively engage in eco-friendly practices across various aspects of their professional roles [43]. This integration encompasses a broad spectrum of activities, from employing sustainable materials and waste reduction techniques in clinical settings to advocating for policies that promote environmental health and sustainability [22]. This educational paradigm shift encourages a holistic view of health that includes the well-being of the planet, ensuring that upcoming nurses are well-prepared to address the challenges of modern healthcare through a sustainability lens [44]. Such an approach not only reinforces the importance of environmental considerations in nursing practice but also positions nurses as key players in leading the transition towards more sustainable healthcare practices globally [45]. This strategic incorporation of sustainability into nursing education underscores the profession’s commitment to fostering a culture of environmental responsibility, ultimately contributing to the development of healthcare professionals who are not just healthcare providers but also guardians of the environment [16]. This reflects the defining attribute of professional development and education, emphasizing continuous learning and the dissemination of sustainable practices.

The concept of eco-conscious nursing extends significantly into the realm of research, serving as a foundational framework that underscores the intricate connections between health and the environment [46]. This research domain is crucial for uncovering innovative and sustainable practices within healthcare, aiming not only to mitigate the sector’s environmental footprint but also to enhance patient care through more holistic and environmentally mindful approaches [47]. Scholars and practitioners alike delve into the study of eco-conscious interventions, ranging from waste reduction and energy efficiency in healthcare facilities to the promotion of green prescribing and the utilization of sustainable medical supplies [48]. Moreover, research in eco-conscious nursing contributes to a broader understanding of how health professionals can act as pivotal agents of change, advocating for policies and practices that protect the environment while promoting public health and wellbeing [49]. Through this lens, eco-conscious nursing research is not just about identifying and implementing green practices but also about reimagining the role of healthcare in fostering a sustainable future [50]. This aligns with the defining attribute of collaboration and leadership, where nurses work alongside other professionals to develop and implement sustainable healthcare solutions.

Eco-conscious nursing, therefore, is not merely about adopting green practices but represents a comprehensive approach to healthcare that prioritizes environmental stewardship as essential to health and wellbeing [51]. Its applications span the spectrum of nursing activities, from direct patient care to community engagement, education, and research, highlighting the profession’s integral role in advancing sustainable healthcare solutions [52].

The defining attributes of eco-conscious nursing—sustainability integration, environmental advocacy, holistic patient care, professional development and education, and collaboration and leadership—are inherently linked to the uses of the concept in various healthcare contexts. These attributes collectively frame eco-conscious nursing as a proactive and engaged approach to nursing, seeking to mitigate environmental harm while promoting a sustainable and healthy future for individuals and communities alike.

### Defining attributes

Defining attributes are essential characteristics that frequently emerge in the literature and are consistently present when a concept manifests [53, 54]. This section aims to identify and elaborate on the critical attributes that define “Eco-conscious Nursing,” underscoring the inherent qualities necessary for this concept’s realization within the nursing and broader healthcare context. Eco-conscious nursing, as illuminated through extensive literature review and conceptual analysis, is characterized by a set of attributes that collectively distinguish it from traditional nursing practices, emphasizing a deliberate focus on sustainability and environmental health.

- **Sustainability Integration**: This attribute involves incorporating sustainability principles into all aspects of nursing care. Eco-conscious nurses actively seek to minimize environmental impact through practices such as waste reduction, energy conservation, and sustainable resource management. This integration requires a conscious effort to balance patient care excellence with environmental stewardship [55].
- **Environmental Advocacy**: Eco-conscious nurses serve as advocates for environmental health within healthcare settings and the wider community. This attribute encompasses raising awareness about environmental determinants of health, advocating for policies that support sustainable healthcare environments, and engaging in community outreach to promote public understanding of environmental health issues [56].
- **Holistic Patient Care**: This attribute reflects a commitment to a comprehensive approach to patient care that includes environmental factors affecting health. Eco-conscious nurses develop care plans considering the physical, psychological, social, and environmental aspects of patient well-being, recognizing the interconnection between human health and the environment [57].
- **Professional Development and Education**: Eco-conscious nurses are committed to continuous learning and professional development related to environmental health and sustainability in healthcare. This attribute includes pursuing education and training opportunities that enhance the ability to incorporate eco-conscious principles into nursing practice and disseminating knowledge about sustainable practices to peers, patients, and the community [58, 59].
- **Collaboration and Leadership** Eco-conscious nursing necessitates collaborative efforts across disciplines and the assumption of leadership roles in initiating and guiding sustainability efforts within healthcare settings. Nurses with this attribute work alongside healthcare professionals, environmental experts, and organizational leaders to develop and implement strategies that reduce the environmental footprint of healthcare operations. [60].

These defining attributes of eco-conscious nursing—sustainability integration, environmental advocacy, holistic patient care, professional development and education, and collaboration and leadership—collectively frame a nursing practice that is attuned to the environmental impacts of healthcare. They underscore a proactive and engaged approach to nursing that seeks not only to mitigate harm but also to promote a sustainable and healthy future for individuals and communities alike. Through the embodiment of these attributes, eco-conscious nurses play a pivotal role in steering the healthcare sector towards more sustainable and environmentally responsible practices.

### Pioneering interventions in other countries

Several countries have spearheaded innovative interventions in eco-conscious nursing, setting exemplary standards for integrating sustainability into healthcare. In Sweden, healthcare facilities have widely adopted energy-efficient lighting and HVAC systems, significantly reducing their carbon footprint. The Swedish approach also includes comprehensive recycling programs and the use of biodegradable medical supplies, showcasing a holistic commitment to environmental sustainability [61]. Similarly, in Australia, hospitals have embraced green building designs that maximize natural light and ventilation, thus cutting down on energy consumption. Initiatives like “Green Health Partnerships” in Australia further promote sustainable practices by educating healthcare professionals and encouraging eco-friendly behaviors within hospitals [62].

In the Netherlands, healthcare institutions have made substantial progress through the implementation of green procurement policies, ensuring that medical supplies and equipment are sustainably sourced [63]. Dutch hospitals also lead in innovative waste management practices, which include the safe disposal and recycling of medical waste. These interventions not only mitigate environmental harm but also foster a culture of sustainability within healthcare settings, positioning these countries as pioneers in eco-conscious nursing [64].

### Concrete research lines

Future research should delve into the impact of eco-conscious nursing on patient outcomes. This includes investigating how sustainable practices influence recovery times, mental health, and overall well-being of patients. Such studies can provide empirical evidence on the benefits of integrating eco-friendly practices in nursing, potentially leading to improved patient care and satisfaction. Another crucial research line involves exploring the economic benefits of sustainable healthcare practices [65]. By analyzing cost savings from reduced energy consumption, efficient waste management, and sustainable procurement, researchers can make a compelling case for the financial viability of eco-conscious nursing.

Additionally, cross-cultural adaptation of eco-conscious nursing practices warrants further investigation. Research should focus on how these practices can be tailored to different cultural contexts to ensure their effectiveness and cultural sensitivity [66]. This can help in developing universally applicable guidelines that respect cultural differences while promoting sustainability. Lastly, the role of policy in advancing eco-conscious nursing practices should be explored. Evaluating existing policies and developing new frameworks can provide insights into the best practices for integrating sustainability into healthcare governance. This includes assessing the impact of policy-driven initiatives on the adoption of eco-conscious practices in various healthcare settings.

### Antecedents and consequences

The exploration of antecedents and consequences is crucial for a comprehensive understanding of “Eco-conscious Nursing,” as it illuminates the factors that foster its emergence and the outcomes that follow its implementation. This analysis aids in delineating the underlying conditions conducive to eco-conscious nursing practices and their subsequent impacts on healthcare and environmental sustainability [67].

### Antecedents of eco-conscious

Nursing Antecedents to eco-conscious nursing are the pre-existing conditions or factors that must be present for the concept to manifest within the healthcare setting. These foundational elements provide the necessary groundwork for the development and recognition of eco-conscious nursing practices [68].

- **Environmental Awareness and Education**: A foundational awareness of environmental issues and a solid educational background in sustainable practices are critical antecedents. Nurses informed about the environmental impact of healthcare operations are better equipped to initiate and participate in sustainable practices [69].
- **Institutional Policies and Support**: Healthcare institutions that prioritize environmental sustainability and support eco-conscious initiatives create a conducive environment for these practices. This support may include providing resources for sustainability projects, implementing green policies, and fostering a culture that values environmental stewardship [65].
- **Interdisciplinary Collaboration**: Collaboration across disciplines is essential for integrating eco-conscious practices into nursing. The synergy between nursing, environmental science, and other healthcare disciplines enhances the development and implementation of sustainable healthcare solutions [70].

### Consequences of eco-conscious nursing

The consequences of eco-conscious nursing refer to the outcomes or effects that arise from the application of sustainability principles within the nursing profession [53]. These effects underscore the impact of eco-conscious nursing on healthcare quality, environmental sustainability, and societal well-being.

- **Improved Environmental Health Outcomes**: Eco-conscious nursing practices can lead to significant improvements in environmental health. By reducing waste, minimizing the use of harmful substances, and conserving resources, nursing practices can directly contribute to reducing the ecological footprint of healthcare facilities [71].
- **Enhanced Patient and Community Well-being**: Sustainable healthcare practices not only have a positive impact on the environment but also on patient and community health. For example, reducing the use of toxic materials in healthcare settings can decrease exposure to harmful chemicals, benefiting both patients and healthcare workers [72].
- **Economic Efficiency**: Implementing eco-conscious practices can result in economic benefits for healthcare institutions. Efficient resource use and waste reduction can lead to cost savings, while sustainable investments can improve long-term financial stability [15].

### **Specific intervention programs**,** their economic impacts**,** and potential challenges and strengths faced by professionals.**

- **Specific Intervention Programs**: Examples of specific intervention programs include the adoption of energy-saving measures such as the utilization of energy-efficient lighting and HVAC systems, which significantly reduce the carbon footprint of healthcare facilities. Another example is the implementation of comprehensive waste management programs that emphasize recycling, proper segregation of waste, and the use of biodegradable products.
- **Economic Impacts**: The economic impacts of these interventions can be substantial. Energy-efficient practices and sustainable waste management can lead to significant cost savings for healthcare facilities. These savings can be reinvested into further sustainability initiatives or other areas of patient care, creating a positive feedback loop that promotes continuous improvement.
- **Challenges and Strengths**: Challenges in implementing these programs often include a lack of awareness, insufficient training in sustainable practices, and entrenched systemic obstacles. However, strengths such as strong institutional support, interdisciplinary collaboration, and ongoing professional development can help overcome these challenges.

These antecedents and consequences collectively frame eco-conscious nursing as a pivotal element in advancing sustainable healthcare. By understanding the conditions that facilitate eco-conscious nursing and recognizing its beneficial outcomes, healthcare professionals and institutions can better integrate these practices into their operations. This integration not only aligns with global sustainability goals but also promotes a holistic approach to health that encompasses both human and environmental well-being.

### Empirical referents

Defining empirical referents for the concept of “Eco-conscious Nursing” entails identifying measurable indicators or observable phenomena that embody this concept [73]. Empirical referents are crucial for operationalizing “Eco-conscious Nursing,” offering a tangible means to evaluate its manifestation and efficacy within healthcare settings [74]. By delineating these referents, researchers and practitioners can objectively measure and assess the presence, implementation, and impact of eco-conscious practices in nursing, facilitating a clearer understanding and fostering the integration of these practices into routine nursing care. The empirical referents for Eco-conscious Nursing might include:

1. Sustainable Waste Management practices:

- **Description**: Sustainable waste management practices include the adoption and effectiveness of waste reduction strategies within healthcare facilities. This can be measured by reductions in medical waste production, increases in recycling rates, and the proper segregation and disposal of hazardous materials.
- **Relation to Eco-Conscious Nursing**: These practices are integral to eco-conscious nursing as they reflect a commitment to minimizing the environmental impact of healthcare operations. Nurses play a crucial role in implementing and advocating for sustainable waste management practices, ensuring that clinical waste is handled in an environmentally responsible manner.

2. Energy Efficiency measures:

- **Description**: Energy efficiency measures refer to the incorporation of strategies and technologies that reduce energy consumption in healthcare settings. This can include the use of energy-efficient lighting, heating, ventilation, and air conditioning (HVAC) systems, as well as the adoption of renewable energy sources.
- **Relation to Eco-Conscious Nursing**: Energy efficiency is a core component of eco-conscious nursing. Nurses contribute to energy-saving efforts by adopting practices that reduce energy use and by promoting the use of energy-efficient technologies. This not only reduces the environmental footprint of healthcare facilities but also demonstrates the nursing profession’s commitment to sustainability.

3. Water Conservation efforts:

- **Description**: Water conservation efforts involve implementing methods to reduce water usage in healthcare operations. This can be assessed by measuring reductions in water consumption, employing water-saving technologies, and promoting water conservation practices among staff and patients.
- **Relation to Eco-Conscious Nursing**: Water conservation is another key aspect of eco-conscious nursing. Nurses can lead initiatives to conserve water in clinical settings, thereby reducing the overall environmental impact and supporting broader sustainability goals.

4. Green procurement policies:

- **Description**: Green procurement policies refer to the adoption of purchasing practices that prioritize eco-friendly products and services. This includes the use of biodegradable disposables, non-toxic cleaning supplies, and sustainably sourced materials.
- **Relation to Eco-Conscious Nursing**: Green procurement is essential for eco-conscious nursing as it ensures that the materials and products used in patient care are environmentally sustainable. Nurses can advocate for and implement green procurement policies to promote sustainability within their healthcare institutions.

5. Nurse-led sustainability initiatives:

- **Description**: The degree to which nurses lead and participate in sustainability initiatives can be an empirical referent. This involvement can be measured by the number and scope of nurse-driven projects aimed at enhancing environmental sustainability, such as educational programs, policy development for reducing carbon footprints, and advocacy for sustainable healthcare practices.
- **Relation to Eco-Conscious Nursing**: Nurse-led initiatives are a direct manifestation of eco-conscious nursing. These projects reflect the proactive role of nurses in promoting environmental health and sustainability, showcasing their leadership and commitment to integrating eco-conscious principles into healthcare practice.

Our analysis suggests that two concepts - sustainable waste management and energy efficiency - are particularly pivotal. These areas not only underscore the practical application of eco-conscious nursing but also highlight its potential to significantly reduce the environmental impact of healthcare operations. Emphasizing these empirical referents enhances the visibility and viability of eco-conscious nursing practices, encouraging their widespread adoption and leading to improved environmental outcomes as well as enhanced patient care and nursing satisfaction.

### Model case

A model case exemplifies a scenario that fully encapsulates all the defining attributes of the concept being analyzed [53]. It serves as an ideal example, illustrating how the concept of eco-conscious nursing can manifest in a real-world setting. In this instance, we will construct a model case to showcase a scenario that encompasses the defining attributes of eco-conscious nursing:

#### Model case

Jasmine represents an exemplary figure in the nursing field, embodying the principles of eco-conscious nursing within a large healthcare facility. Her journey in nursing has been marked by a steadfast commitment to integrating environmental sustainability into healthcare practices. Jasmine has pursued additional certifications in environmental health and sustainability, showcasing her dedication to eco-conscious principles. As a leader in her facility, she has spearheaded initiatives aimed at reducing waste, conserving energy, and promoting sustainable resource use in patient care practices.

In her role, Jasmine exercises a significant level of professional autonomy and authority, allowing her to implement innovative eco-friendly practices. She has been instrumental in transitioning her department to the use of biodegradable materials and ensuring the adoption of energy-efficient medical devices. Her efforts have not only reduced the environmental impact of her facility but also served as a cost-saving measure, showcasing the dual benefits of eco-conscious nursing practices.

Jasmine’s influence extends beyond her immediate responsibilities. She is an active member of the hospital’s sustainability committee, where she collaborates with other healthcare professionals to develop hospital-wide policies that enhance environmental sustainability. Her contributions are highly valued, and she plays a critical role in decision-making processes related to sustainability initiatives. Through her leadership, Jasmine has fostered a culture of eco-consciousness within her team, encouraging continuous education on environmental health issues and sustainable practices.

Moreover, Jasmine leverages her access to resources and information to stay abreast of the latest developments in sustainable healthcare. She regularly attends workshops and seminars on environmental sustainability and shares this knowledge with her colleagues, enhancing their capacity to contribute to eco-friendly initiatives. Her commitment to professional development in the realm of eco-conscious nursing is evident in her advocacy for incorporating sustainability principles into nursing curricula and continuing education programs.

As a respected figure in her healthcare facility and the broader nursing community, Jasmine has garnered recognition for her pioneering work in eco-conscious nursing. She serves as a role model and mentor to aspiring nurses interested in sustainability, exemplifying how dedication to eco-conscious principles can lead to meaningful changes in healthcare practices. Her case embodies the essence of eco-conscious nursing, illustrating the profound impact that nurses can have on promoting environmental sustainability in healthcare settings. Jasmine’s story is a testament to the power of nursing professionals to effect change, not only in patient care but also in leading the way towards a more sustainable future in healthcare.

### Borderline case

Borderline cases exemplify scenarios that demonstrate some, but not all, attributes of eco-conscious nursing practices [75]. These instances help refine and delineate the concept by showcasing examples that are on the fringe or exhibit only partial aspects of eco-consciousness in nursing. The following outlines a borderline case:

#### Case

Max is a nurse in a suburban community hospital striving to incorporate eco-conscious practices into his daily routines. While he lacks the authority to implement wide-scale environmental policies or access to a broad array of eco-friendly supplies, he demonstrates a commitment to sustainability within his scope of influence. Max advocates for reducing single-use plastic usage in his department by encouraging the use of reusable alternatives where possible and consistently educates his patients and colleagues about the environmental impacts of healthcare waste. However, the hospital’s limited budget for sustainability initiatives and the absence of a formal eco-conscious framework hinder his ability to fully integrate comprehensive green practices into patient care and departmental operations.

In this borderline case, Max exhibits key attributes of eco-conscious nursing, such as a commitment to sustainability and proactive engagement in eco-friendly practices. However, he encounters significant constraints, including limited institutional support and resources, which curtail the full expression of eco-conscious nursing as defined by broader, systemic changes and adoption of sustainability measures. Despite these challenges, Max’s efforts to incorporate environmental considerations into his nursing practice underscore the nuanced manifestations of eco-conscious nursing. His case illustrates that while individual actions are valuable, the realization of eco-conscious nursing on a larger scale requires structural support and resources.

This borderline scenario emphasizes the complexity of eco-conscious nursing, showcasing how nurses can embody the spirit of sustainability even when systemic barriers exist. It highlights the need for healthcare institutions to provide support and resources that enable nurses like Max to fully engage in eco-conscious practices. Examining cases like Max’s broadens our understanding of eco-conscious nursing, acknowledging the spectrum of its application and the varied contexts in which it can occur. It also points to the importance of institutional policies and support in facilitating the transition towards more sustainable healthcare practices.

#### Contrary case

A contrary case for the concept of eco-conscious nursing illustrates a scenario that starkly contrasts with the core attributes of eco-conscious practices in nursing [73]. Such a case helps delineate the concept’s boundaries and clarifies what it does not encompass. For instance, consider the situation of Maya, a nurse working in a healthcare setting that places minimal emphasis on environmental sustainability. Despite awareness of the environmental impact of healthcare waste and energy use, the facility where Maya works lacks recycling programs, continues to use single-use disposable materials extensively, and has no initiatives in place to reduce energy consumption. Maya herself does not prioritize or engage in sustainable practices, either due to a lack of knowledge, interest, or support from her workplace. She rarely considers the environmental impact of her actions or the healthcare services provided, focusing solely on immediate patient care needs without regard to long-term environmental consequences.

In this contrary case, Maya’s disengagement from eco-conscious practices and her workplace’s indifference to sustainability starkly oppose the defining characteristics of eco-conscious nursing. The absence of initiative to minimize environmental impact, along with a lack of policies supporting sustainability, reflects a complete negation of eco-conscious principles. Furthermore, Maya’s lack of involvement in and advocacy for sustainable practices contrasts sharply with the proactive and committed stance integral to eco-conscious nursing. This scenario underscores the absence of awareness, motivation, and institutional support as key barriers to embedding eco-consciousness in nursing practice.

Examining Maya’s case sheds light on the importance of individual and organizational commitment to environmental sustainability in healthcare. It highlights how the absence of eco-conscious principles in nursing can lead to missed opportunities for reducing healthcare’s environmental footprint and promoting a healthier planet. Moreover, this case serves as a reminder of the need for education, policy development, and leadership in fostering eco-conscious nursing practices. By understanding what eco-conscious nursing is not, through the lens of this contrary case, the distinct and essential features of the concept become more pronounced, guiding efforts to integrate sustainability more deeply into the nursing profession and healthcare at large.

## Discussion

This study endeavors to unravel the concept of “Eco-conscious Nursing,” by delineating its key attributes, antecedents, and consequences. Our primary goal is to establish an operational definition that is both pertinent and resonant across the myriad settings where eco-conscious nursing finds relevance. Through a detailed analysis, we aim to uncover the nuanced layers of eco-conscious nursing and its broad implications within the healthcare landscape. Central to our discussion is the recognition of eco-conscious nursing as a fluid and evolving construct, shaped by factors such as environmental awareness, sustainable healthcare practices, and the integration of green principles into nursing care. This adaptability underscores the necessity for a sophisticated grasp of the concept, encouraging its integration into academic debates and practical healthcare applications alike. The proposed operational definitions are crafted with contextual versatility in mind, presenting adaptable insights for both scholarly and practical environments.

In our quest to define “Eco-conscious Nursing” through its attributes, antecedents, and consequences, it’s vital to formulate a precise operational definition. This definition encapsulates the core characteristics, precursors, and impacts of eco-conscious nursing, enhancing the concept’s clarity and application.

- **Comprehensive operational definition of Eco-conscious Nursing** Eco-conscious Nursing is identified as a professional ethos within healthcare, characterized by a commitment to environmental sustainability and the application of eco-friendly practices in nursing care. This ethos empowers nurses with the knowledge and practices necessary for minimizing environmental impact, fostering a culture of sustainability within healthcare settings. Antecedents include a foundational understanding of environmental health and sustainability principles, while consequences manifest as enhanced patient and community health outcomes, alongside reduced ecological footprints of healthcare practices.

Given the intricate and multi-faceted nature of “Eco-conscious Nursing,” we have expanded the operational definition to encapsulate critical components that imbue the concept with its significance and applicability in both academic and operational spheres. We identified two crucial dimensions: environmental stewardship, as reflected in the stewardship-centric operational definition, and sustainable practice, highlighted in the practice-focused operational definition. Thus, in addition to the comprehensive definition, we present two more operational definitions tailored to the specific scope and context of eco-conscious nursing’s application.

- **Stewardship-centric operational definition** Eco-conscious Nursing represents a professional stance wherein nurses champion environmental stewardship, advocating for and implementing sustainable practices within healthcare. It involves the authority to influence healthcare policies and practices towards sustainability, autonomy in integrating eco-friendly practices, and gaining respect for such initiatives. Antecedents are rooted in a strong educational foundation in environmental health, leading to outcomes that significantly improve the sustainability of healthcare services and patient care through efficient resource use and sustainable practices.
- **Practice-focused operational definition** Eco-conscious Nursing signifies a professional approach characterized by the practical implementation of sustainable healthcare practices. It grants nurses the autonomy to apply eco-friendly practices, access to sustainable resources, and the authority to influence patient care and operational policies. Founded on a robust educational background in sustainability and environmental health, its consequences include heightened professional satisfaction and an uplifted standard of care that aligns with environmental sustainability goals. This definition emphasizes the importance of practical, sustainable actions and decision-making in nursing, marking a shift towards more environmentally responsible healthcare practices.

These operational definitions reflect the depth and breadth of eco-conscious nursing, emphasizing its pivotal role in advancing sustainable healthcare practices. They serve not only as a conceptual framework for further academic exploration but also as a practical guide for integrating sustainability into nursing practice, ultimately leading to a healthcare system that is both ethically responsible and environmentally sustainable.

### Future implications

The exploration and conceptual analysis of “Eco-conscious Nursing” set forth in this study open several avenues for future research, practice development, and policy formulation, each holding significant potential to advance the integration of sustainability within the nursing profession and the broader healthcare sector. The implications of this study extend into various dimensions, including educational curricula, clinical practice, healthcare policy, and global health initiatives, underlining the pivotal role of nursing in spearheading sustainable healthcare solutions.

### Educational curricula

Integrating eco-conscious principles into nursing education is essential for preparing future nurses to engage in sustainable practices. Nursing programs should incorporate environmental sustainability as a core competency, equipping students with the knowledge and skills to implement eco-friendly practices in their professional roles. This shift in educational focus will ensure that upcoming nurses are well-prepared to lead the transition toward more sustainable healthcare systems.

### Clinical practice

The practical applications of eco-conscious nursing in various healthcare settings highlight a roadmap for integrating sustainable practices into daily nursing care. Future research should explore innovative strategies for reducing waste, conserving energy, and promoting eco-friendly patient care practices. Developing best practice guidelines based on the findings of this study can facilitate the widespread adoption of eco-conscious practices, enhancing the environmental sustainability of healthcare operations.

### Healthcare policy

The conceptual analysis presented here lays a foundation for advocating policy changes at institutional, national, and international levels. Policymakers should consider the operational definitions and frameworks proposed in this study to craft legislation and regulations that support sustainable healthcare practices. Policies that incentivize eco-friendly initiatives and embed environmental stewardship into healthcare governance will be crucial for promoting eco-conscious nursing.

### Global health initiatives

The study’s findings align with global health priorities such as the United Nations Sustainable Development Goals (SDGs). Leveraging the concept of eco-conscious nursing to address global health challenges emphasizes the role of nurses in implementing sustainable health interventions. Collaborative international research and cross-sector partnerships can further elucidate the global impact of eco-conscious nursing practices, contributing to a healthier planet and populations.

#### Innovation and leadership

The future of eco-conscious nursing requires innovation in practice and leadership in sustainability initiatives. Nurses equipped with the insights from this study are poised to take leadership roles in sustainability efforts within healthcare settings. Future research should focus on identifying and overcoming barriers to implementing eco-conscious practices, developing leadership programs for nurses in environmental health, and fostering a culture of innovation that encourages sustainable healthcare solutions.

In conclusion, the implications of this study are far-reaching, offering a blueprint for integrating eco-conscious principles into the nursing profession and healthcare at large. By advancing education, practice, policy, and global health initiatives focused on sustainability, the nursing profession can significantly contribute to the creation of a more sustainable and health-focused future.

### Limitations

This study, while pioneering in its effort to elucidate the concept of Eco-conscious Nursing, encounters several limitations that merit consideration. First and foremost, the scope of literature reviewed may not encompass all existing material pertinent to eco-conscious nursing practices. Despite a rigorous and systematic approach to the literature search, the possibility remains that certain relevant studies, particularly those published in languages other than English or in less accessible journals, were not included. This limitation could potentially narrow the breadth of perspectives and evidence integrated into our analysis.

Furthermore, the conceptual analysis method, while robust in its ability to dissect and clarify complex concepts, relies heavily on the subjective interpretation of the researchers. This subjectivity may influence the selection of attributes, antecedents, and consequences deemed significant to the operational definitions of eco-conscious nursing. Although efforts were made to mitigate bias through methodical analysis and peer consultation, inherent subjectivity cannot be entirely eliminated.

## Conclusion

This concept analysis of Eco-conscious Nursing provides a foundational understanding of integrating environmental sustainability principles into nursing practice. By delineating key attributes, antecedents, and consequences, this study underscores the critical role of nurses in promoting sustainable healthcare practices. Eco-conscious nursing is characterized by sustainability integration, environmental advocacy, holistic patient care, professional development, and collaboration. These attributes collectively frame a nursing practice that addresses the environmental impacts of healthcare, positioning nurses as essential agents of change.

### Global health priorities and SDGs

The findings resonate with global health priorities, particularly the United Nations Sustainable Development Goals (SDGs). Eco-conscious nursing aligns with SDG 3 (Good Health and Well-being), SDG 6 (Clean Water and Sanitation), SDG 7 (Affordable and Clean Energy), SDG 12 (Responsible Consumption and Production), and SDG 13 (Climate Action). By promoting environmentally sustainable practices, nurses contribute to better health outcomes, responsible resource management, and climate change mitigation.

### Future directions

This study lays the groundwork for future research on the empirical application of eco-conscious nursing practices, exploring their economic and cross-cultural dimensions. Developing best practice guidelines and policy frameworks will facilitate the widespread adoption of eco-conscious nursing, ensuring sustainability becomes integral to healthcare delivery.

In conclusion, integrating eco-conscious principles into nursing aligns with global sustainability goals and empowers nurses to lead towards a more sustainable and ethically responsible healthcare system. As the concept of eco-conscious nursing evolves, it holds the potential to significantly impact the healthcare sector and the broader global community, fostering a healthier and more sustainable future for all.

## Data Availability

The datasets generated during and/or analyzed during the current study are available from the corresponding author on reasonable request.
