# Correction: Advancing sustainable healthcare: a concept analysis of eco-conscious nursing practices

**DOI:** 10.1186/s12912-024-02433-7

**Published:** 2024-10-21

**Authors:** Marwa Mamdouh Shaban, Majed Awad Alanazi, Huda Hamdy Mohammed, Fatma Gomaa Mohamed Amer, Hla Hosny Elsayed, Mohammed ElSayed Zaky, Osama Mohamed Elsayed Ramadan, Mohamed Ezzelregal Abdelgawad, Mostafa Shaban

**Affiliations:** 1 Faculty of Nursing, Cairo University, Cairo, Egypt; 2 College of Nursing, Jouf University, Sakaka, Saudi Arabia; 3 Faculty of Nursing, Ain Shams University, Cairo, Egypt; 4 Faculty of Nursing, Alexandria University, Alexandria, Egypt

**BMC Nursing (2024) 23:660**

10.1186/s12912-024-02197-0

The original article erroneously presented co-author, Osama Mohamed Elsayed Ramadan’s name which has since been corrected.

